# The significance of metalloproteinase 3 (MMP-3), chemokine CXC ligand 13 (CXCL-13) and complement component C5a in different stages of ANCA associated vasculitis

**DOI:** 10.1038/s41598-021-84662-3

**Published:** 2021-03-04

**Authors:** Aleksandra Rymarz, Magdalena Mosakowska, Stanisław Niemczyk

**Affiliations:** Department of Internal Diseases, Nephrology and Dialysis, Military Institute of Medicine, 128 Szaserów Street, 04-141 Warsaw, Poland

**Keywords:** Kidney diseases, Diagnostic markers, Rheumatic diseases

## Abstract

The aim of the study was to evaluate the significance of metalloproteinase 3 (MMP-3), chemokine CXC ligand 13 (CXCL-13) and complement component 5a (C5a) in different stages of ANCA associated vasculitis (AAV). 89 adults were included into the study. 28 patients with active AAV (Birmingham Vasculitis Activity Score, BVAS > 3) formed the Active Group. 24 individuals who were in remission after 6 months of induction therapy formed the Short R Group, while 34 patients with longitudinal remission formed the Long R Group. 28 patients without autoimmune diseases similar in terms of age, gender and stage of kidney disease formed the Control Group. Receiver operating characteristic curve analysis (ROC) was used to evaluate MMP-3, CXCL-13 and C5a as markers of the different phases of vasculitis. In ROC analysis, MMP-3, CXCL-13 and C5a presented a good ability in distinguishing active vasculitis (Active Group) from the Control Group (AUC > 0.8), whereas only CXCL-13 displayed potential ability in distinguishing active vasculitis (Active Group) from long term remission (Long R Group, AUC = 0.683). MMP-3 significantly and positively correlated with serum creatinine concentration (r = 0.51, *p* = 0.011; r = 0.44, *p* = 0.009; r =  −0.66, *p* < 0.001) and negatively with eGFR (r =  −0.5, *p* = 0.012; r =  −0.35, *p* = 0.039; r =  −0.63, *p* < 0.001) in the Short R, Long R and Control Groups. MMP-3, CXCL-13, C5a can be potential markers in differentiating an active phase of vasculitis from other pathologies. However they can be treated as complementary to the well-known markers. CXCL-13 seems to be a potential marker in distinguishing active vasculitis from long term remission. MMP-3 level can be related to kidney function expressed by eGFR, therefore its elevation should be interpreted with caution in patients with kidney failure.

## Introduction

According to the International Chapel Hill Consensus Conference (CHCC2012), anti-neutrophil cytoplasmic autoantibody (ANCA)-associated small vessel vasculitides (AAV) consist of three different diseases: granulomatosis with polyangiitis (GPA), microscopic polyangiitis (MPA), and eosinophilic granulomatosis with polyangiitis (EGPA, Churg–Strauss syndrome)^1^. The presence of specific autoantibodies are present in many of them. GPA is related to the presence of proteinase 3 (PR3), which is detected in 90% of patients with generalized GPA and in 50% of patients with localized form of GPA^2^. Myeloperoxidase (MPO) is found in 70% of patients with MPA and in 35% of patients with EGPA^3,4^. The availability of antibody detection tests in recent years has enabled a quicker and easier diagnosis of vasculitis, however, in some cases differentiating between reactivation and remission can pose a challenge. Therefore, searching for new markers specific to vasculitis is extremely important.

AAV are diseases which affect small vessels such as small arteries, arterioles, capillaries, venules, and sometimes medium-sized arteries and veins^5^. ANCA activate neutrophils and monocytes as well as the alternative complement pathway^6^. Consequently infiltrations comprising leukocytes cause the destruction and necrosis of the vessels’ wall as well as occlusion of the vessels’ lumen, thus organs supported by these vessels become ischemic and damaged. In the inflammatory environment around the vessel wall, there are many proteolytic enzymes which directly cause destruction of the vessels. These enzymes can be markers of inflammatory activity. One of them is metalloproteinase 3 (MMP-3) also referred to as stromelysin-1. It can degrade many components of basement membranes and connective tissue such as collagen types II, IV, IX and fibronectin, laminin, proteoglycans, elastin^7^. MMP-3 can activate other metalloproteinases and pro-inflammatory mediators^8^. This enzyme was identified in both the glomerular and tubular cells of the kidney^8^.

The chemokine CXC ligand 13 (CXCL-13), also named B-cell-attracting chemokine-1 (BAC-1) or B-lymphocyte-chemoattractant (BLC), is a CXC subtype member of the chemokine superfamily. CXCL-13 is involved in B cell attraction and the stimulation of intracellular pathways thus inducing cell invasion, survival and growth^9^. New studies proved the role of neutrophil extracellular traps (NET) in AAV pathogenesis. The study made by Kraij S et al. showed that NET formation was excessive in active phase of the disease and is independent form ANCA titre. The NETs also activated autoreactive B cells^10^. Monach et al. highlighted MMP-3 and CXCL-13 as good markers for distinguishing an active form of AAV from remission^11^.

In the past the role of complement in the pathogenesis of AAV was considered to be insignificant mostly because of lack of complement components in kidney biopsies. Recent data showed that alternative pathway of complement activation has a crucial role in the development of active AAV. ANCA bind to neutrophil’s surface, activate them and stimulate releasing of many factors including properdin which stimulates the activation of alternative pathway of complement. During this process anaphylatoxin C5a is released and amplifies the neutrophil activation through the receptor CD88. Recent studies showed that C5a were elevated in serum and urine of patients with active AAV^12^.

Three markers associated with tissue destruction, B-lymphocytes chemoattraction and with an alternative pathway complement activation were chosen to analysis. The aim of the study was to evaluate the significance of MMP-3, CXCL-13 and C5a in different stages of AAV such as the active phase, short-term remission, long-term remission and comparing with patients without any autoimmune disorders.

## Methods

### Study population

89 adults were included into the study. They were recruited between November 2015 and March 2018. To assess the activity of AAV the BVAS v3 (Birmingham Vasculitis Activity score version 3) and BVAS/WG were used^13,14^. Patients with clinically active ANCA-associated vasculitis (GPA and MPA) and a BVAS of at least 3 formed the first group (Active Group). Individuals who after 6 months of induction therapy had reached remission—defined as BVAS 0—formed the short term remission group (Short R Group). The following group consisted of patients with remission of ANCA-associated vasculitis for more than 6 months and no shorter than 1 year after the beginning of the induction therapy, treated with supportive therapy (Remission Group). The final group was the control group (Control Group) which consisted of individuals without autoimmune disease, similar in terms of age, gender and the stage of kidney disease to the Active Group.

The diagnosis of ANCA-associated vasculitis was based on the criteria established by the American College of Rheumatology in 1990 and Revised International Chapel Hill Consensus Conference Nomenclature of Vasculitides—CHCC2012^1,15^.

Exclusion criteria were as follows: vasculitis without ANCA, lack of patient compliance.

The protocol of the study was accepted by the Local Ethics Committee. Written informed consent was signed by all study participants.

### Laboratory measurements

Blood samples in the Active Group were taken before the beginning of induction immunosuppressive treatment and in hemodialysis patients before the dialysis session. The plasma was separated within 30 min and samples were taken for measuring MMP-3 (matrix metalloproteinase-3), CXCL-13 (chemokine (C-X-C motif) ligand 13), C5a (complement component 5a) and they were kept frozen at − 80 °C. ANCA (antineutrophil cytoplasmic antibodies) were measured using Enzyme-Linked Immunosorbent Assay (ELISA) with fluorochrome. The cut-off point for positive values was > 5.0 IU/ml for MPO ANCA (antineutrophil cytoplasmic antibodies against myeloperoxidase) and > 3.0 IU/ml for PR3 ANCA (antineutrophil cytoplasmic antibodies against proteinase 3). MMP-3, CXCL-13, C5a were measured using ELISA tests (*Human MMP-3 Platinum ELISA*, eBioscience; *BLC/CXCL13 Human ELISA Kit*, Thermo scientific; *Human C5a Platinum ELISA Kit*, eBioscience).

Serum creatinine concentrations, serum albumin (bromocresol purple), serum prealbumin were measured by routine methods at the local Department of Laboratory Diagnostics. Estimated glomerular filtration rate (eGFR) was calculated on the basis of short MDRD formula.

### Statistical analysis

Continuous variables were summarized using descriptive statistics (mean and standard deviation or median and interquartile range). Categorical variables were calculated as percentages. To check the normality of the distribution of variables, the Kolomogorov–Smirnov test was used. The differences between the groups were assessed using the Kruskal–Wallis test. Correlations between kidney function and biochemical markers were calculated using Spearman's rank-order correlation coefficient. To assess the power of a marker in distinguishing the different phases of vasculitis, a receiver operating characteristic curve analysis (ROC) was used. Cut-off points were calculated using Youden's index. The characteristics of selected markers were presented as an area under the curve with 95% confidence interval, sensitivity, specificity. The level of statistical significance of tests used was set at *p* < 0.05. The analysis was performed using the Statistica12 package StatSoft Poland.

### Ethics approval

This study was performed in line with the principles of the Declaration of Helsinki. Approval was granted by the Ethics Committee of Military Institute of Medicine, Warsaw, Poland (18.06.2014/No. 26/WIM/2014).

### Consent to participate

Informed consent was obtained from all individual participants included in the study.

### Consent for publication

There is not identifying information or images about the patients included in the article.

## Results

28 patients with severe, active AAV were included into the Active Group (BVAS/WG 8.46 ± 3.66). 24 patients formed the short term remission group (Short R Group) and 34 formed the long term remission group (Long R Group). The groups did not differ in terms of age and gender. Among the organs frequently affected were the joints, lungs and kidneys. Cyclophosphamide was the main drug used in the induction therapy which was associated with a restricted usage of rituximab in the country where the study was undertaken. In patients from the control group presented chronic kidney disease, the underlying diseases were as follows: atherosclerosis (17.8%), hypertensive nephropathy (17.8%), polycystic kidney disease (14.8%), nephrectomy (10.7%), obstructive nephropathy (7.1%), kidney agenesis (7.1%), Alport’s syndrome (3.5%), gout nephropathy (3.5%), unknown (7.1%).

Clinical characteristics of the studied groups are presented in Table 1.

**Table 1:** Clinical characteristics of the study population.

	Active Group (N = 28)	Short R Group (N = 24)	Long R Group (N = 34)	Control Group (N = 28)
Age, mean ± SD, years	58.32 ± 15.14	57.4 ± 15.5	61.00 ± 12.86	60.3 ± 16.8
Gender, female/male (%)	19 (67.9%) / 9 (32.1%)	17 (70%) / 7 (30%)	21 (61.8%) / 13 (38.2%)	18 (66.6%) / 9 (33.3%)
ANCA PR3/MPO	17 (71%) / 11 (29%)	14 (58%) / 10 (42%)	22 (65%) / 12 (35%)	–
Clinical diagnosis GPA/MPA	16(57%)/12(43%)	13(54%) / 11(56%)	23(68%) /11(32%)	–
BVAS v3, mean ± SD	19.14 ± 7.14	0	0	–
BVAS/WG mean ± SD	8.46 ± 3.66	0	0	–
**Organ involvement**				
Skin	7 (25%)			
Joints	24 (86%)			
URT	15 (54%)			
Lungs	24 (86%)			
kidneys/HD	21 (75%) / 12(43%)			
CNS	7 (25%)			
**Induction therapy**				
GCS	28 (100%)	24 (100%)		
Cyclophosphamide	18 (64%)	15 (63%)		
Rituximab	6 (22%)	6 (25%)		
Others	4 (14%)	3 (12%)		
**Maintenance therapy**				
GCS		24 (100%)	32 (94%)	
AZA		14 (58%)	13 (40%)	
MMF		7 (29%)	14 (44%)	
Others		1(4%)	9 (28%)	

*GCS* glucocorticosteroids, *GPA* granulomatosis with polyangiitis, *MPA* microscopic polyangiitis.

Among laboratory parameters serum creatinine concentration did not differ among the groups, however, in the Active Group it was higher in comparison than in the Short R Group, though the difference was on the border of statistical significance (*p* = 0.051). Daily proteinuria was significantly lower in the Long R Group when compared to the Active Group (*p* = 0.012). Erythrocyturia was at its highest in the Active Group and decreased significantly in the Short R and Long R Groups. Laboratory parametres in four studied groups are presented in Table 2.

**Table 2:** Laboratory findings in the study groups.

	Active Group	p*	Short R Group	p**	Long R Group	Control Group	p***
SCr, mean, mg/dl	3.39 ± 2.21	0.051	2.86 ± 2.05	0.664	2.59 ± 2.12	4.05 ± 3.51	0.427
eGFR	33.17 ± 30.46	0.054	37.87 ± 31.11	0.502	41.70 ± 30.2	33.6 ± 33.3	0.436
Proteinuria, mean, g/24 h	1.07 ± 1.5	0.275	0.75 ± 0.93	**0.012**	0.51 ± 1.22	0.88 ± 1.66	0.425
Erythrocyturia > 4 HPF	81.9%	** < 0.001**	18%	** < 0.001**	9%	19%	** < 0.001**
ANCA Median (IQR)	51.0 (14.9–137.5)	** < 0.001**	4.5 (2.3–21.5)	**0.001**	2.6 (0.4–36)	0.2 (0.1–0.25)	** < 0.001**
CRP, Median (IQR) mg/dl	2.0 (0.4–7.1)	** < 0.001**	0.2 (0.1–0.4)	** < 0.001**	0.2 (0.1–0.8)	0.3 (0.15–0.25)	** < 0.001**
MMP-3 Median (IQR) ng/ml	123.6 (85.5–248.1)	0.239	163.4 (121.1–293.6)	0.950	134.4 (74.8–218.0)	58.1 (45.5–89.4)	** < 0.001**
CXCL-13 Median (IQR) pg/ml	198.0 (162.3–347.1)	0.397	184.7 (107.9–427.2)	**0.013**	145.7 (79.2–216.8)	102.4 (61.4–131.3)	** < 0.001**
C5a Median (IQR) ng/ml	0.2 (0.196–0.329)	0.162	0.2 (0.157–0.257)	0.218	0.2 (0.115–0.314)	0.1 (0.07–0.14)	** < 0.001**

*ANCA* anti-neutrophil cytoplasmic autoantibody, *CRP* C-reactive protein, *CXCL-13* chemokine CXC ligand 13, *HPF* high power field, *MMP-3* metalloproteinase 3, *SCr* serum creatinine concentration.

p* for comparison of Active Group with Short-term remission Group, p** for comparison of Long-term remission Group with Active Group, p*** for comparison of Control Group with Active Group.

*p* values which are statistically significant are bolded.

ANCA titers were at the highest levels in patients from the Active Group. They were significantly higher in comparison to the Short R, Long R and Control Groups.

CRP levels were also significantly higher in the Active Group in comparison to the Short R, Long R and Control Groups.

MMP-3 levels were significantly higher in all groups of patients who suffered from vasculitis in comparison to the Control Group. However, the levels did not differ between the Active Group and Short R Group or between the Active Group and Long R Group. CXCL-13 levels were also significantly higher in all groups of patients who suffered from vasculitis in comparison to the Control Group. Among the patients in the Long R Group, levels of CXCL-13 were significantly lower than among those in the Active Group (*p* = 0.013). C5a complement component levels were significantly lower in the Control Group in comparison to the Active or Short R and Long R Groups.

**Figure 1:**
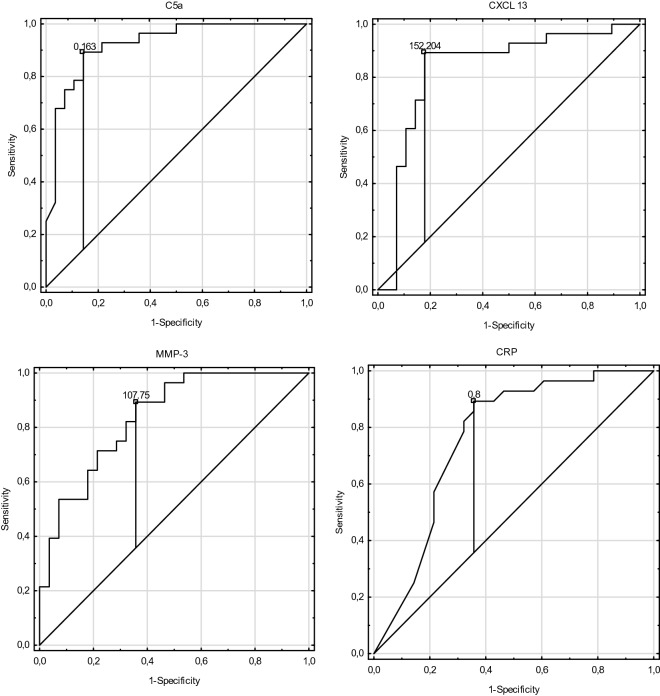
Receiver operating characteristic curve for C5a, CXCL-13, MMP-3 and CRP showing the ability of markers to distinguish active vasculitis (Active Group) from the control group with cut-off points.

**Figure 2:**
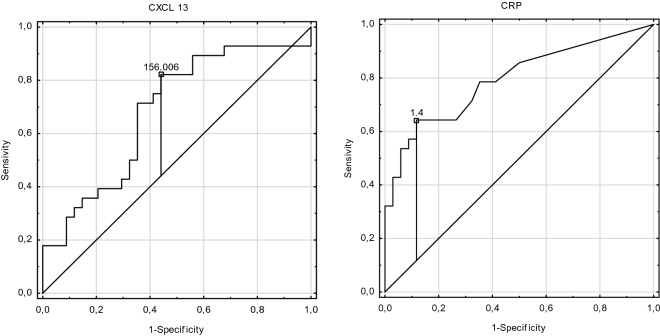
Receiver operating characteristic curve for CXCL-13 and CRP showing the ability of markers in distinguishing active vasculitis (Active Group) from long term remission (Long R Group) with cut-off points.

Receiver operating characteristic curve analysis (ROC) revealed a good ability of MMP-3, CXCL-13 and C5a in distinguishing active vasculitis (Active Group) from the Control Group (AUC > 0.8), (Fig. 1; Table 3). Whereas only CXCL-13 had the potential ability to distinguish active vasculitis (Active Group) from long term remission (Long R Group, AUC > 0.8), (Fig. 2; Table 4).

**Table 3:** Receiver operating characteristic curve analysis for C5a, CXCL-13, MMP-3 and CRP comparing the Active Group with the Control Group.

	Active Group versus Control Group			
	AUC	SE	95% CI	Cut-off point
CRP	0.782	0.067	0.631–0.894	0.8 mg/dl
MMP-3	0.838	0.052	0.736–0.94	107.75 ng/ml
CXCL-13	0.832	0.061	0.712–0.951	152.2 pg/ml
C5a	0.923	0.036	0.854–0.993	0.16 ng/ml

AUC, area under the curve; CRP, C-reactive protein; CXCL-13, chemokine CXC ligand 13; C5a, complement component C5a; MMP-3, metalloproteinase 3; SE, standard error.

**Table 4:** Receiver operating characteristic curve analysis for CXCL-13 and CRP comparing the Active Group with the Long R Group.

	Active Group versus Long R Group			
	AUC	SE	95% CI	Cut off point
CRP	0.794	0.059	0.679–0.909	1.4
CXCL-13	0.683	0.069	0.547–0.818	156

*AUC* area under the curve, *CRP* C-reactive protein, *CXCL-13* chemokine CXC ligand 13, *SE* standard error.

Spearman’s rank-order correlation analysis was performed to examine the association between renal function expressed by serum creatinine concentration (SCr) or eGFR and the inflammatory markers which were studied. A significant positive correlation was observed between SCr and CRP in the Active Group (0.46; *p* = 0.012) and the Control Group (0.38; *p* = 0.042). Furthermore, a significant and positive correlation was observed between SCr and MMP-3 in the Short R (0.51; *p* = 0.011), Long R (0.44; *p* = 0.009) and Control Group (0.66; *p* < 0.001) (Table 5).

**Table 5:** Correlations between serum creatinine concentration or eGFR and inflammatory markers in the studied groups.

	Active Group	Short R Group	Long R Group	Control Group
**SCr**				
CRP	0.46	0.31	0.12	0.38
	*p* = 0.012	*p* = 0.073	*p* = 0.560	*p* = 0.042
MMP-3	0.01	0.51	0.44	0.66
	*p* = 0.950	*p* = 0.011	*p* = 0.009	*p* < 0.001
CXCL-13	0.027	0.26	0.28	0.34
	*p* = 0.891	*p* = 0.206	*p* = 0.105	*p* = 0.074
C5a	0.15	0.37	0.08	− 0.05
	*p* = 0.442	*p* = 0.070	*p* = 0.642	*p* = 0.794
**eGFR**				
CRP	− 0.50	− 0.09	− 0.284	− 0.38
	*p* = 0.007	*p* = 0.649	*p* = 0.104	*p* = 0.043
MMP-3	0.02	− 0.50	− 0.35	− 0.63
	*p* = 0.906	*p* = 0.012	*p* = 0.039	*p* < 0.001
CXCL-13	− 0.091	− 0.32	− 0.32	− 0.37
	*p* = 0.647	*p* = 0.120	*p* = 0.060	*p* = 0.05
C5a	− 0.13	− 0.39	− 0.07	0.07
	*p* = 0.500	0.059	*p* = 0.669	*p* = 0.723

## Discussion

The aim of our study was to compare new markers of vasculitis activity in different phases of ANCA-associated small vessel vasculitis. MMP-3 as a marker of tissue destruction, CXCL-13 as a chemoattractant factor, C5a as a marker of complement activation and CRP as a general marker of inflammatory process were chosen.

In the presented study, median MMP-3 levels did not differ significantly between groups with vasculitis, neither with the active nor the short or long-term remission groups. However, MMP-3 levels were significantly lower in the control group in comparison to the groups with vasculitis (*p* < 0.001). The ROC curve analysis revealed that MMP-3 displayed a good ability in distinguishing between those patients in the active phase of the disease from those without autoimmune disorders (AUC > 0.838; Table 3; Fig. 1). The cut-off point for MMP-3 was at the level of 107.7 ng/ml. Similar results were described by Zakiyanov et al.^16^ who did not observe a difference between MMP-3 levels in active and remission groups of patients with AAV, whereas the results of the study performed by Monach et al., which included 137 patients with active AAV, revealed a significant decline in MMP-3 in patients with short-term remission in comparison to active patients^11^. The same researchers also observed a significant difference between MMP-3 levels in healthy subjects compared to patients with an active phase of AAV. An interesting finding in our study, which to the best of our knowledge, had not thus far been described, was the significant correlation between kidney function and the MMP-3 level in the three studied groups: short-term remission, long-term remission and control groups. As in other proinflammatory markers, the MMP-3 level can also be dependent on kidney function and may express a subclinical, low-grade inflammatory state which is present in chronic kidney disease patients^17^. The lack of a significant correlation between MMP-3 and kidney function in patients with active disease can be attributed to the fact that high-grade inflammation, which accompanies active vasculitis, overlaps on slightly expressed inflammation resulting from CKD. Levels of other markers such as CXCL-13 and C5a did not correlated with kidney function but they are responsible for different actions than MMP-3 in the inflammatory process.

CXCL-13 is a chemokine which is involved in B cell attraction to the inflammatory area. Schiffer et al. observed increased CXCL-13 levels accompanying B cell infiltrations in kidney biopsies in patients with a T cell mediated rejection of transplanted kidneys^18^. B cells also play an important role in the pathogenesis of AAV^19^. Therefore, CXCL-13 is examined as a potential vasculitis biomarker. In our study, the median levels of CXCL-13 were at their highest level in the active group and decreased gradually in the following groups: short-term remission, long term remission and the control group. The differences reached statistical significance between the active group and the long-term remission group (*p* = 0.033) and between the control and active groups (*p* < 0.001) as well as the short-term remission group (*p* < 0.001). The explanation of this finding can be associated with B cells activity associated with different phases of vasculitis. The lowest concentration of CXCL-13 in long term remission Group (Long R Group) can reflect deep B cell depletion in this group. In the study performed by van Dam et al. in patients with PR-3 ANCA vasculitis treated with rituximab most of the relapses (81%) were associated with B cells regeneration and B cell status together with ANCA titer were predictive factors for the relapses^20^ Vasculitis is a group of diseases which are relapsing, but differentiating between the early phase of the relapse and remission is often very difficult. Moreover, non-specific inflammatory markers such as erythrocyte sedimentation rate (ESR) or C-reactive protein (CRP) levels can be elevated during the relapse of the AAV and also during infection complications of AAV treatment. Therefore, a marker which is non-invasive and specific for vasculitis activation process is to be expected. In the study published by Monach et al. CXCL-13 was a good marker in distinguishing between active vasculitis from remission or from healthy subjects^11^. In our study we observed that the CXCL-13 level was significantly lower in the long-term remission group in comparison to the active group but we did not observe a significant difference between the active and short-term remission groups. This phenomenon can be associated with B lymphocytes which are affected by immunosuppression in the longer term.

AAV as an inflammatory process is associated with an increased level of non-specific parameters such as CRP. In our study CRP levels were significantly higher in the active group than in the short-term remission group (*p* < 0.001), the long-term remission group (*p* < 0.001) as well as in the control group (*p* < 0.001). ROC curve analysis revealed that AUC for CRP was above 0.7 in distinguishing the Active Group from the Long Remission or Control Group. The cut-off for distinguishing the active phase in long-term remission for CRP was 1.4 mg/dl, whereas for distinguishing active vasculitis from other diseases was 0.8 mg/dl. Similar results were obtained by Monach et al.^11^.

Activation of the complement pathway in the pathogenesis of AAV is crucial. C5a, which is a component of the alternative pathway together with ANCA, stimulate neutrophils and the coagulation system represented by thrombin generation^21^. In our study, levels of C5a were significantly higher in the active group than in the control group (*p* < 0.001). ROC curve analysis revealed that it can be a good parameter in distinguishing the active phase of vasculitis from other pathologies (AUC 0.923).The cut-off point was at a level of 0.16 ng/ml.

The studies on complement involvement in AAV resulted in the synthesis of a selective inhibitor of C5a receptor which is referred to as avacopan. In a randomized, placebo-controlled trial, avacopan allowed the replacement of high doses of glucocorticosteroids^22^.

The results of our study did not show a significant superiority of one marker over the other. Three proposed markers represent different actions in the pathogenesis of AAV. They can be treated as a complementary to well known markers such as CRP, ESR or ANCA. Each of them characterize different phases of inflammatory process. Therefore diagnosis based on few complementary parameters could help to establish the stage of the diseases.

In summary, MMP-3, CXCL-13, C5a can be helpful in differentiating an active phase of vasculitis from other pathologies. However they can be treated as complementary to the well-known markers. CXCL-13 seems to be a potential marker for distinguishing active vasculitis from long term remission. The MMP-3 level can be related to kidney function expressed by eGFR, therefore its elevation should be interpreted with caution in patients with kidney failure.
